# Diagnostic needs for rare diseases and shared prediagnostic phenomena: Results of a German-wide expert Delphi survey

**DOI:** 10.1371/journal.pone.0172532

**Published:** 2017-02-24

**Authors:** Susanne Blöß, Christian Klemann, Ann-Katrin Rother, Sandra Mehmecke, Ulrike Schumacher, Urs Mücke, Martin Mücke, Christiane Stieber, Frank Klawonn, Xiaowei Kortum, Werner Lechner, Lorenz Grigull

**Affiliations:** 1 Department of Pediatric Hematology and Oncology, Hannover Medical School, Hannover, Hannover, Germany; 2 Center for Chronic Immunodeficiency (CCI), University Medical Center Freiburg, University of Freiburg, Freiburg, Germany; 3 Center for Pediatric and Adolescent Medicine, University Medical Center Freiburg, University of Freiburg, Freiburg, Germany; 4 Department of Neurology, Hannover Medical School, Hannover, Germany; 5 DRK Clementinenkrankenhaus, Hannover, Germany; 6 Center for Rare Diseases Bonn (ZSEB), University Hospital of Bonn, Bonn, Germany; 7 Ostfalia University of Applied Sciences, Wolfenbuettel, Germany; 8 Helmholtz Centre for Infection Research, Braunschweig, Germany; 9 Improved Medical Diagnostics IMD GmbH, Hannover, Germany; Sant Joan de Déu Children's Hospital, SPAIN

## Abstract

**Background:**

Worldwide approximately 7,000 rare diseases have been identified. Accordingly, 4 million individuals live with a rare disease in Germany. The mean time to diagnosis is about 6 years and patients receive several incorrect diagnoses during this time. A multiplicity of factors renders diagnosing a rare disease extremely difficult. Detection of shared phenomena among individuals with different rare diseases could assist the diagnostic process. In order to explore the demand for diagnostic support and to obtain the commonalities among patients, a nationwide Delphi survey of centers for rare diseases and patient groups was conducted.

**Methods:**

A two-step Delphi survey was conducted using web-based technologies in all centers for rare diseases in Germany. Moreover, the leading patient support group, the German foundation for rare diseases (ACHSE), was contacted to involve patients as experts in their disease. In the survey the experts were invited to name rare diseases with special need for diagnostic improvement. Secondly, communal experiences of affected individuals were collected.

**Results:**

166 of 474 contacted experts (35%) participated in the first round of the Delphi process and 95 of 166 (57%) participated in the second round. Metabolic (n = 74) and autoimmune diseases (n = 39) were ranked the highest for need for diagnostic support. For three diseases (i.e. scleroderma, Pompe’s disease, and pulmonary arterial hypertension), a crucial need for diagnostic support was explicitly stated. A typical experience of individuals with a rare disease was stigmatization of having psychological or psychosomatic problems. In addition, most experts endured an ‘odyssey’ of seeing many different medical specialists before a correct diagnosis (n = 38) was confirmed.

**Conclusion:**

There is need for improving the diagnostic process in individuals with rare diseases. Shared experiences in individuals with a rare disease were observed, which could possibly be utilized for diagnostic support in the future.

## Introduction

In Europe a disease is considered rare when it affects less than 1 in 2,000 individuals. Approximately 13.5 million Europeans are affected with 1 of 7,000 known rare diseases (RD) [1] and 4 million Germans have a RD. RDs also called orphan diseases cover a wide spectrum of disorders. Some RDs are apparent at birth; however, most are discovered after a long period of searching and coping by afflicted individuals [2]. Persons with a RD might not feel sick, but rather just different, which hinders the diagnosis and consequently leads to delays in diagnosis [2, 3]. This diagnostic delay—which is even more pronounced in RDs affecting multiple organs—, sometimes on the order of years, frequently results in inappropriate treatments or missed treatment opportunities and is associated with increased morbidity or mortality [2–5].

As illustrated in patients with achalasia, the children were inaccurately diagnosed and mistreated for more common medical conditions and in some patients the duration of symptoms before the diagnosis of achalasia was 6 to 10 years [6]. Likewise, diagnostic delays characterize other RDs. In patients with primary ciliary dyskineasia (PCD) the median age at diagnosis was 4.4 years in a majority of patients [7]. The prolong time period before the diagnosis of PCD led to severe chronic and irreversible lung damage [8, 9]. For individuals with late-onset Pompe’s disease, there may be many years from first symptom to diagnosis [10]. During this period without a proper diagnosis, patients were wrongly identified as having chronic fatigue or obesity and the benefits of enzyme replacement therapy were markedly postponed. Patients with an inborn immune dysfunction also suffered from diagnostic delays. Here, the prediagnostic time was typified by severe and sometimes life-threatening and debilitating infections [11].

In general, progressive RDs—such as metabolic diseases where slow accumulation of a substance leads to unspecific symptoms or slowly degenerative neurological RDs pose an enormous diagnostic challenge and the entry point is pivotal for success or failure of the diagnostic process.

Traditionally, the general practitioner (GP) is the gatekeeper to detect a RD in many instances. Becoming familiar and identifying all RDs, however, is unrealistic for most physicians including the GP. Nevertheless, the GP has the important job of initiating or recommending further evaluation(s) for the patients with (a suspected) RD. Only when the GP considers a RD can a referral to an expert be initiated. Therefore, the GP should be supported in this critical triaging process [3]. Ideally, the GP should refer patients with an unusual constellation of symptoms or insufficient response to therapy to a center of expertise covering a broad range of rare diseases. Today, this process is not well established.

To overcome these shortcomings experts established a European strategic plan and national strategic plans to improve the care for individuals with a RD, including training and awareness activities [5,6]. According to the European Union Committee of Experts on rare diseases (EUCERD) one priority area in the field of RD must be the diagnosis [12,13]. Some RDs are obvious early in life (e.g. omphalocele, gastroschisis) and here the focus is on therapy and consultation with the family. Many other RDs present with nonspecific symptoms which are often regarded as a ‘personal feature’ of the patient rather than a diagnostic clue. In these cases, diagnostic support has the potential to shorten the diagnostic latency and improve outcomes for patients. In order to identify the challenges of diagnosing a RD, we conducted a German-wide Delphi survey among centers for rare diseases (CRD) (n = 24) and RD patient groups to include patients serving as experts in their disease. The Delphi process was chosen for integrating and merging a wide spectrum of expert opinions. We asked which RDs require the utmost diagnostic support. This question was raised not for prioritization but to gain insight into the diagnostic needs. Furthermore, we hypothesized that experts in RDs might be able to denominate commonalities of individuals with different RDs. This hypothesis is based on the fact that individuals with a RD feel ‘different’ from their peers in certain aspects of daily life. We asked the experts to share subjective and objective events and experiences, termed phenomena, among RD patients.

Thus, the aims of the study were to define commonalities in patients with different rare diseases during their prediagnostic journey and to determine in a Delphi survey which RDs need diagnostic support.

## Materials and methods

### Delphi survey

The Delphi method is an established method to achieve a convergence of opinion among a group of experts. The technique, originally developed by the RAND Corporation, utilizes a series of questionnaires administered in sequential rounds. Questions are presented, answered and the answers are analyzed to generate directed re-queries which allow for revision of initial answers by participants. The process is repeated until a consensus answer is achieved [14]. The Delphi technique was developed to overcome problems associated with freely interacting groups such as dominant individuals and pressure to conform to the majority viewpoint [15]. The survey is regarded as the optimal method for systematic collection of opinions on a predefined topic [16]. Due to anonymization of the survey, peer pressure is avoided [17].

In September 2014, 24 German CRDs and five European institutions (Zurich, Basel, Innsbruck, Padua, and London) specializing in RDs were invited via email to participate in an online query. The head of the German foundation for individuals with RDs (ACHSE, alliance of chronic and rare diseases/conditions) was also invited to involve patients who are experts in their disease. Therefore both medical professional and patient inputs were guaranteed. The query consisted of two questions. First, ‘Please specify 3 to 5 rare diseases, where you think that diagnostic support is of utmost importance.’ Second, ‘According to your personal experience, what are shared phenomena in individuals with a rare disease?’

**Fig 1:**
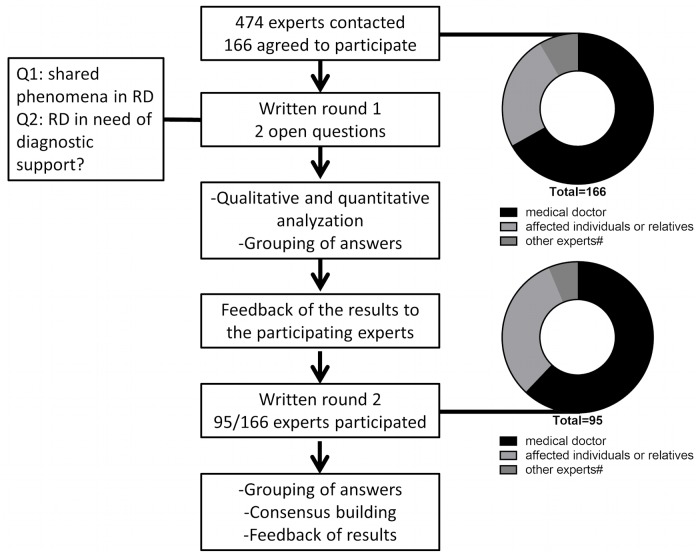
The Delphi process is illustrated. In the Delphi survey, a total of 474 experts were contacted initially. During the second round, 95 participated. All participants were invited to answer two questions.

In the first round of the Delphi survey, 474 RDs experts (for details see Table 1) were asked to name diseases where diagnostic support is needed and to specify commonalities among individuals affected with different RDs. In the second Delphi round, all experts who participated in the first round were contacted again. The results of the first round were briefly represented and the experts were asked to (again) prioritize RDs for diagnostic support. The final votes were clustered in disease groups according to ICD-10 codes. The diseases were then ranked by the number of votes. In the second round, 95 of 166 (57%) experts participated and again identified the diseases or disease groups that need diagnostic support. In total, the 95 experts named 266 diseases or disease groups. The results are listed in Tables 2 & 3. The shared phenomena of individuals with a RD were only requested in one round (Fig 1). Case studies were created in close cooperation with affected individuals to illustrate the pre-diagnostic time. In Fig 1, the Delphi process is illustrated.

**Table 1:** Background of participants responding to the first Delphi inquiry.

Profession	Replies on 1st survey (n)	Replies on 2nd survey (n)
Medical doctor	111	59
Affected individual or relatives	41	30
Other experts*	14	6
Total	166	95

* e.g. nurses, psychologists, and biologists

**Table 2:** First 20 diseases with utmost need for diagnostic support identified in the first Delphi round*.

Rare disease	Number of times named by experts (n)
Progressive sclerodermia	10
Pompe’s disease	7
Pulmonary arterial hypertension (PAH)	7
Systemic lupus erythematosus (SLE)	7
Cystic fibrosis (CF)	6
Mediterranean fever	6
Morbus Fabry (M. Fabry)	6
Amyotrophic lateral sclerosis (ALS)	5
Severe combined immunodeficiency (SCID)	5
Leukodystrophy	4
McArdle’s disease	4
Myelodysplastic syndrome	4
Morbus Wilson (M.Wilson)	4
Chronic granulomatous disease (CGD)	4
Ataxia telangiectasia	3
Cluster headaches	3
Ehlers-Danlos syndrome (EDS)	3
Morbus Hurler	3
Niemann-Pick Type C	3
Ornithine transcarbamylase (OTC) deficiency	3

*Hashimoto thyroiditis was mentioned but omitted because not fulfilling the criteria for a rare disease.

**Table 3:** Rare diseases with need for diagnostic support (2nd Delphi round).

Disease group	Disease specification	Disease name
Metabolic disease (n = 74)	Metabolic disease storage type (n = 24)	Mucopolysaccharidosis (n = 6)
		Pompe’s disease (n = 8)
		M. Fabry (n = 6)
	Metabolic disease ‘intoxication type’ (n = 13)	Urea cycle defects (n = 5), OTC deficiency (n = 2)
	Glycogenosis (n = 6)	McArdle’s disease (n = 4)
	Metabolic disease with hepatic manifestation (n = 4)	M. Wilson (n = 4)
Autoimmune diseases (n = 39)	Collagenosis (n = 11)	Scleroderma (n = 5)
	Autoinflammatory diseases (n = 7)	Fever syndromes (n = 6)
Neuromuscular diseases (n = 28)		Amyotrophic lateral sclerosis (ALS) (n = 3)
Primary immune deficiencies (n = 27)		Severe combined immune deficiency (SCID) (n = 10)
Rare cancer syndromes (n = 20)		Myelodysplastic syndrome (n = 2)
Rare pulmonary diseases (n = 12)		Primary pulmonary arterial hypertension (n = 6)
		Cystic fibrosis (n = 3)
Pain syndromes (n = 9)		Cluster headache (n = 5)
Rare hematological diseases (n = 8)		Fanconi anemia (n = 2)
Rare diseases of the eyes (n = 8)		Glaucoma in children (n = 2)
Diseases with psychomotor abnormalities (n = 7)		Ataxia telangiectasia (n = 2)
Rare diseases with endocrinological manifestation (n = 6)		Acromegaly (n = 2)
Rare diseases of the soft tissue (n = 5)		Ehlers-Danlos syndrome (n = 4)

### Analysis of the data

We conducted both a qualitative and quantitative analysis of the answers. All answers collected during the Delphi surveys were processed and analysed with MAXQDA 11. The software facilitates selection of codes and assigns answers to categories [18]. After completion of the survey, units of significance were calculated. Then by using MAXQDA 11, a code was assigned to each paragraph. Subsequently, related codes were placed in comparable categories. The categories were similarly compared and reorganized until (a) theme(s) emerged. To ensure reliability of the data, the findings were verified by the participants, researchers, and two external reviewers [18].

## Ethical consideration

The Ethics Committee of the Medical University of Hannover approved the conduct of the study, and written informed consent was obtained from all participants (Approval number: 2316–2014). For children, the informed written consent was provided by the legal guardians.

## Results

### Case studies to highlight the diagnostic journey of individuals with a RD

#### Patient 1

A 36 years old male reported frequent medical consultations during childhood due to decreased performance during sport activities and poor endurance. In addition, he experienced nausea during school sport. He was considered ‘weak’ and in need of more training/exercise. Due to his poor endurance he never learned to swim and hiking was often interrupted prematurely. Pain following physical exertion was deemed as muscle aches. Despite his limitations, he joined the military service where he was exempt from sport and marching activities. Later he became a heating engineer, but he required assistance for ‘energy-sapping’ tasks. Only during an evaluation for kidney stones were his elevated creatine kinase levels noted and further workup revealed a diagnosis of McArdle’s disease.

#### Patient 2

A 53 years old female patient began to notice changes to her physical appearance ten years prior to her diagnosis. Rings did not fit her fingers anymore and her nose, ears and mandible grew and her teeth shifted progressively. Most distressing was new onset hirsutism. She also developed blisters from walking in her old beloved hiking shoes. Ashamed of her appearance, the patient withdrew from social activities. She sought consultation with different medical professionals (e.g. GP, dentist, physiotherapist, employee health) due to her diverse symptoms, but no one considered a common link between her complaints. She was evaluated by an otolaryngologist for snoring. She visited her GP and subsequently a rheumatologist and various orthopedic surgeons for ankle pain. Surgery was completed for hallux rigidus and trigger digits and she also received bilateral hip replacement. During her odyssey, buying new shoes become impossible and daily activities such as holding a cup of coffee were painful tasks. Only after ten years, did a new rheumatologist consider acromegaly based on her appearance and the diagnosis was subsequently confirmed.

### Distribution of experts participating

In total, 166 of 474 (35%) experts replied to the first round of the Delphi inquiry. Participants were mostly medical doctors (n = 111) and affected individuals or relatives (n = 41) (Table 1).

### Diseases with special need for diagnostic improvement

456 diseases or disease groups were identified to be in need of diagnostic support. Of these diseases, 284 could be grouped into five categories (metabolic diseases, autoimmune conditions, neuromuscular diseases, primary immunodeficiencies, and rare types of cancer). More than 10 experts identified 380 of the 456 diseases. Here, rare pulmonary diseases (e.g. cystic fibrosis, primary pulmonary hypertension), endocrinological disorders (e.g. acromegaly), skin diseases, and hematological disorders as well as pain syndromes were selected (Table 2). Diseases with high priority for diagnostic support selected in the first Delphi round are listed in Table 2. Here, Progressive sclerodermia, Pompe’s disease, Pulmonary arterial hypertension (PAH), Systemic lupus erythematosus (SLE), Cystic fibrosis (CF), Mediterranean fever, Morbus Fabry, Amyotrophic lateral sclerosis (ALS) and severe combined immunodeficiency (SCID) were frequently named.

In the second Delphi round the votes of the experts were asked to opt again for diseases where diagnostic support is highly warranted. Both, diseases and disease groups were named by the experts. Metabolic diseases were specified by the experts (e.g. Pompe’s disease, mucopolysaccaridosis, M. Fabry) followed by auto-immune diseases (e.g. collagenosis, fever syndromes). Out of this second Delphi round a list of diseases with high need for diagnostic support was created. Of note, immune deficiencies, neuromuscular diseases and rare cancer syndromes also received high votes in terms of need for diagnostic support.

### Commonalities of individuals with a RD

All participants were also invited to identify shared phenomena among individuals with a RD according to his/her personal experience as an expert.

In our survey 373 different commonalities were detected during the first survey, which were then grouped into five categories (Table 4). 183 quotations were grouped into the category of “Peculiar emotional experiences and perceptions of individuals with a rare disease” and 80 quotations belong into the category “The odyssey of going to many different doctors and receiving different diagnoses” (Table 4).

**Table 4:** Categories of shared phenomena in individuals with a rare disease.

Category	(n)	Themes	Example/citations from participants
Peculiar emotional experiences and perceptions of individuals with a rare disease	183	Self-doubt, frustration, and/ordepression	‘I had self-doubt’; ‘I had an overwhelming feeling that there was something wrong’; ‘I thought there was something funny with me’; ‘Patients have a high degree of suffering.’
The odyssey of going to many different doctors and receiving different diagnoses	80	Long journey,Odyssey	‘The diagnostic odyssey’; ‘When doctors can’t find the proper diagnosis they tend to say it’s a psychological thing’; ‘In women with a rare disease, some doctors blame the hormones and neglect other possible diagnoses’
Diagnostic challenges in rare diseases, the issue of misdiagnosis and misunderstanding	63	Rare diseases are not considered, lack of classical symptoms makes the diagnosis even more challenging; patients are not taken seriously	‘Symptoms were considered as being of psychological in nature’;‘Doctors lacked time for proper clinical reasoning’;‘Doctors never took my health complaints seriously’; ‘Some doctors worked single-mindedly towards the goal of confirming a wrong diagnosis’
Deficiencies in the health system	28	No contact person, no network in the healthcare system, no diagnostic pathway for individuals without firm diagnosis	
Treatment and therapy	19	Unnecessary surgery or no improvement after surgery or medical treatment, frequent consultations	

### Analysis of commonalities

Emotional perceptions and/or experiences of individuals with a RD prior to diagnosis were ranked highest, noted 183 times during the survey. Important statements in this cluster of entries were ‘*Nobody believed me*’ or ‘*I was sent from one doctor to the next*, *but nobody put the symptoms together*!’ (Table 4). Some experts stated that individuals with a RD were frequently labelled as ‘*dissembler’*. The second most frequent commonality encountered in the survey was a prolong time period to establish a correct diagnosis. Some experts called this period a ‘*diagnostic journey*’ and 42 participants noted this was typical for individuals affected by a RD (Table 4). Furthermore, 14 participants detailed that individuals with a RD received false psychological diagnoses or incorrect somatic diagnoses up to six occasions.

In the cluster of health system-related aspects, ‘*Lack of time of the doctor*’ and ‘*Lack of careful history taking and thorough clinical reasoning*’ were mentioned. The physicians were regarded as constantly being overtaxed. Furthermore, experts reported that in female patients with RDs, doctors attributed the clinical symptoms exclusively to menopause to the detriment of omitting other diagnoses.

## Discussion

This survey found commonalities among different RDs during the prediagnostic time and experts see the need for improving the diagnosis in certain RDs. According to various national action plans for patients with RDs, there is a clear need for improving the diagnostic process of these disease entities [12]. Due to the rarity of some disorders, nonspecific or variability of symptoms, and a multitude of other factors, the diagnosis of a RD is not easy and often belated. Some RDs have distinct biochemical tests and physicians ‘simply’ need to think of the RD and perform the test, whilst in other RDs the challenge is to think of it and to diagnose it in the absence of a specific test (e.g. ALS). Even among experts for RDs, providing the correct diagnosis of a RD can be challenging. Often experts—including RD experts—interpret symptoms in the context of their speciality. Similar to our case study 2, Prencipe et al. described patients with acromegaly and despite comorbidities associated with acromegaly each symptom was treated in isolation. No one was cognisant of the underlying systemic disease and thus the mean time to diagnosis was 5 to 8 years [19]. In another example, although patients with Morquio A syndrome were evaluated by experts in inherited diseases of metabolism, the initial diagnoses were incorrect [20]. Consequently, many RD patients and families take the initiative to determine the cause of their symptomology. Bouwman et al. reported the diagnostic odyssey in patients with rare metabolic diseases [21]. Only after searching the internet of the symptoms of their 11 years old child, was the diagnosis of Morbus Fabry suspected after 6 years of symptoms [21].

Clearly improved approaches to facilitate diagnosis are needed for patients with RDs. The process cannot be simplified for every RD but important strides have been achieved for some rare disorders. Today, many individuals with inborn errors of metabolism (IEM) are detected through national screening programs. Nonetheless, those patients with diseases not included in screening initiatives are overlooked and in clinical practice, their diagnosis frequently needs a long time of searching. We aspire to develop a ‘warning system’ to alert the treating physician, particularly the GPs who are gatekeepers, of a possible diagnosis of a RD when a patient exhibits a pattern consistent with a RD. This approach proved useful for children with rare pulmonary diseases, such as PCD or cystic fibrosis and a logical stepwise evaluation could be initiated to establish differential diagnoses and a final diagnosis [22].

To this end, we hypothesized that knowledge of common phenomena among individuals affected by a RD might give clue to the presence of a RD and shorten the diagnostic delay that characterize these disorders. This study aimed at identifying commonalities in individuals with a RD from the point-of-view of experts using the Delphi process which proved useful in the context of RDs [23]. To the best of our knowledge, there has been no previous study designed to assist the diagnosis of a RD. Patients with different RDs harbor similarities which may be incorporated into a diagnostic process. The surveys conducted in this study revealed two novel results. First, experts were able to prioritize RDs for which diagnostic support is particularly needed which may be important to future diagnostic development. Second, individuals with different RDs share common experiences during the prediagnostic time, which itself is an indication of a RD.

Concerning these commonalities, 183 experts mentioned that ‘peculiar emotional phenomena’ were prevalent in individuals with a RD in the prediagnostic period. ‘Frustration’ and ‘self-doubt’ described the emotional state of many RD individuals who felt ‘different’ or ‘unwell’, but medical professionals could not explain their symptoms. Interestingly, similar results have been reported previously [24]. In a study from Australia, the median time from onset of symptoms to diagnosis of muscular dystrophy was 7.1 years [24]. Parents and patients described ‘stress’, ‘frustration’, and/or ‘anxiety’ during the time before reaching a definitive diagnosis. Other families with Duchenne described an odyssey of seeing one specialist after another without receiving a proper diagnosis. According to our data, this ‘peculiar emotional phenomenon’ was not limited to families with Duchenne but was a feature of many affected by different RDs. Similar phenomena were observed in individuals with Ehlers-Danlos syndrome (EDS). Here, the variable clinical presentation and lack of a molecular confirmatory test contributed to the diagnostic difficulties [25]. Stillmore, some patients appeared clinically normal [25] and the diagnosis of EDS was delayed up to 10 years [26,27]. Indeed, many experts in our survey highlighted the problem of diagnosing a disease in an individual who looked well. This particularity of feeling unwell by patients, yet no one else recognizes the problem(s), is subsumed in the category of ‘diagnostic challenges’ in our analysis.

These results reveal two important ‘red flags’ to the medical professional that a RD may be present. First, the peculiar emotion of ‘frustration’ or ‘feeling different’ during the prediagnostic period was common in patients with RDs. Second, the diagnostic process was impeded by ‘well appearance’ of the patients or absence of typical symptoms, which also produced ‘misdiagnosis and misunderstanding’ and consequent labelling of patients as having psychiatric or psychosomatic problems. In particular, individuals with helicobacter infections, chronic urticaria, or tropical infections are at risk of being misdiagnosed with psychiatric and/or psychosomatic disorders [28–30]. Adding to the complexity of the issue, adolescents with IEM or heterozygous carriers of the disorder might decompensate later in life with dementia or depression and metabolic diseases are not considered in patients with these psychological symptoms [31,32-]. Thus it is tempting to speculate that recurrent and/or acute episodes of psychiatric symptoms might be a symptom of an IEM or another RD. Ahrens-Nicklas et al. emphasized that unexplained episodic fever, decompensation during stressful periods, failure to thrive, avoidance of certain foods, and/or past family history of unexplained early childhood death should trigger further diagnostic for IEM [33]. Certainly, there are potential devastating consequences of an undiagnosed IEM underscoring urgency of diagnosing an IEM at the earliest moment [34]. Likewise, delays in diagnosis of other RDs have negative consequences in both the short- and long-term. In patients with mevalonate kinase deficiency, the median time to diagnosis was 7.1 years and some patients were hospitalized more than 10 times prior to the diagnosis [31], receiving unnecessary or even harmful tests and medications.

The aspect of ‘failure to improve after surgery or medical treatment’ among individuals with RDs was also observed by the experts in our study. Sixty-three experts stated that this phenomena was typical for RDs patients and another 19 mentioned commonalities regarding the therapy in individuals with a RD. Unfortunately, this resulted in doctors not taking patients’ complaints seriously and again, erroneously marking the complaints as psychiatric or psychosomatic in etiology. Based on our data, we advocate that RDs should be considered in ‘psychiatric and/or psychosomatic’ patients not responsive to appropriate therapy [33, 4].

One limitation of our study might be the selection of German-speaking experts and institutions covering only a selection of RD. Therefore, it is unclear whether diagnostic needs might reflect the local situation and not hold for other countries. However, according to the results published by Linertová et al. our results are consistent with other European experiences [23]. Besides, it might be criticised that the term ‘diagnostic support’ used in the survey is only a vague term which differs between different RDs.

RDs are diagnostically difficult. We identified RDs in need of diagnostic support and shared prediagnostic phenomena among RD patients that physicians including the GP should consider as a clue to the possibility of a RD. In a patient whose symptoms are not improving despite appropriate therapy, visiting many doctors, and/or where symptoms do not appear to fit together, the medical practitioner should thoroughly review the medical history, undertake a careful clinical examination, and seriously contemplate a RD diagnosis with referral to the appropriate RD specialist in a timely manner.

## Conclusions

Diagnostic support is needed for most individuals with a RD. Experts considered diagnostic support particularly necessary for patients with metabolic, autoimmune, neuromuscular disorders and rare cardiopulmonary diseases. Our data indicate that patients with a RD are different compared to other patients. Among patients with various RDs, however, they share prediagnostic phenomena. These common experiences include a high degree of frustration due to lack of a definitive diagnosis, seeing various doctors, that something is wrong despite misgivings from medical professionals, and inaccurately being branded as having psychological problems. These typical pattern of a ‘diagnostic journey’ before diagnosis might serve as a base for developing diagnostic support tools for doctors.

## Abbreviations

ACHSE: German foundation for rare diseasesartificial neural network
ALS: Amyotrophic lateral sclerosis
CDDS: clinical decision support systems
CF: cystic fibrosis
CGD: chronic granulomatous disease
CRD: Center for rare disease
EDS: Ehlers-Danlos syndrome
EUCERD: European Union Committee of Experts on rare diseases
GP: General practitioner
IEM: Inborn error of metabolism
OTC: Ornithine transcarbamylase
PAH: Primary arterial hypertension
PCD: primary ciliary dyskinesia
RD: rare disease
SCID: severe combined immune deficiency
SLE: systemic lupus erythematodes
